# The Role of the T2–FLAIR Mismatch Sign as an Imaging Marker of IDH Status in a Mixed Population of Low- and High-Grade Gliomas

**DOI:** 10.3390/brainsci10110874

**Published:** 2020-11-19

**Authors:** Eftychia Z. Kapsalaki, Alexandros G. Brotis, Alexandra Tsikrika, Christos Tzerefos, Thanos Paschalis, Efthymios Dardiotis, Konstantinos N. Fountas

**Affiliations:** 1 Department of Diagnostic Radiology, General University Hospital of Larissa, Faculty of Medicine, University of Thessaly, 41-334 Larissa, Greece; ekapsal@med.uth.gr; 2 Department of Neurosurgery, General University Hospital of Larissa, 41-334 Larissa, Greece; chris.tzefos@gmail.com (C.T.); thanospaschalis@gmail.com (T.P.); 3 Department of Diagnostic Radiology, General University Hospital of Larissa, 41-334 Larissa, Greece; altsikrika@hotmail.com; 4 Department of Neurology, General University Hospital of Larissa, Faculty of Medicine, University of Thessaly, 41-334 Larissa, Greece; ebsdar@gmail.com; 5 Department of Neurosurgery, General University Hospital of Larissa, Faculty of Medicine, University of Thessaly, 41-334 Larissa, Greece; fountas@uth.gr

**Keywords:** T2–FLAIR mismatch sign, glioma, diagnosis, prediction

## Abstract

Our study evaluated the role of the T2–fluid-attenuated inversion recovery (FLAIR) mismatch sign in detecting isocitrate dehydrogenase (IDH) mutations based on a mixed sample of 24 patients with low- and high- grade gliomas. The association between the two was realized using univariate and multivariate logistic regression analysis. There was a substantial agreement between the two raters for the detection of the T2–FLAIR mismatch sign (Cohen’s kappa coefficient was 0.647). The T2–FLAIR mismatch sign when co-registered with the degree of tumor homogeneity were significant predictors of the IDH status (OR 29.642; 95% CI 1.73–509.15, *p* = 0.019). The probability of being IDH mutant in the presence of T2–FLAIR mismatch sign was as high as 92.9% (95% CI 63–99%). The sensitivity and specificity of T2–FLAIR mismatch sign in the detection of the IDH mutation was 88.9% and 86.7%, respectively. The T2–FLAIR mismatch sign may be an easy to use and helpful tool in recognizing IDH mutant patients, particularly if formal IDH testing is not available. We suggest that the adoption of a protocol based on imaging and histological data for optimal glioma characterization could be very helpful.

## 1. Introduction

Gliomas constitute the most common primary brain tumors and are characterized by their infiltrative behavior into the surrounding white matter [1,2]. They represent a very diverse group of neoplasms with variable outcomes [2,3]. Glioma grading is important in the therapeutic management of these patients regarding planning of the optimal surgical approach, the extent of tumor removal, their overall therapeutic management, and their prognosis [1,2]. Accordingly, the 2016 WHO classification of CNS tumors required the molecular analysis of a number of foci, including the isocitrate dehydrogenase (IDH) 1/2 gene and T53 mutation status, the 1p/19q codeletion, and the O^6^-methylguanine-DNA methyl-transferase (MGMT) gene methylation for proper classification of gliomas [2]. There is an increasing body of evidence that mutations at these sites constitute powerful prognostic markers concerning both progression-free and mean survival [2,4,5,6,7,8,9,10]. However, knowledge of the gene status is important preoperatively, and molecular analysis is not ubiquitous and not always feasible.

**Figure 1:**
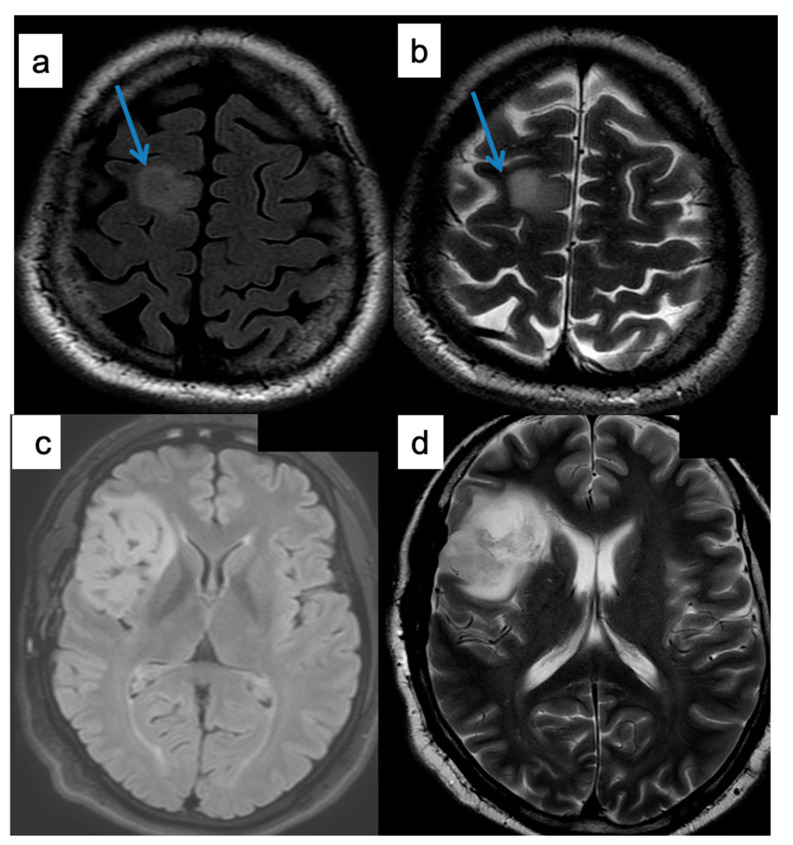
T2–FLAIR mismatch sign. (**a**,**c**) are fluid-attenuated inversion recovery (FLAIR) images while (**b**,**d**) are T2 weighted images (WI).

Recent advances in glioma MRI imaging suggest that the identification of the so called “T2–FLAIR mismatch sign” seemed to be an accurate marker of 1p/19q status among IDH mutant lower grade gliomas (LGG), thus identifying those gliomas with better prognosis (Figure 1) [11,12,13,14,15]. The T2–fluid-attenuated inversion recovery (FLAIR) mismatch sign is present when a lesion is characterized by a hyperintense signal in the T2 weighted images (WI) and a hypointense signal in the FLAIR sequence [16]. It actually reflects differences in the relaxation time characteristics of the IDH mutant and IDH wild-type LGG [17]. A histopathological study showed that the T2-FLAIR mismatch sign might reflect microcyst formation in IDH mutant astrocytomas and could be common in IDH mutant protoplasmic astrocytomas [18]. Therefore, in the absence of molecular analysis, the use of the T2–FLAIR mismatch sign, along with histologic assessment, could optimize diagnosis [15].

Figure 1 a and b (top row) show a T2–FLAIR mismatch sign, since the central part of the tumor has low signal intensity (SI) on FLAIR and high SI on T2 images. Figure 1 c and d (bottom row) do not show a T2–FLAIR-mismatch sign, since the lesion appears with a high signal in both sequences.

Therefore, the T2–FLAIR mismatch sign could also have an important role in predicting the IDH status of gliomas, which may alter the extent of surgical removal of a glioma [16,19]. The purpose of our current study was to evaluate the presence of the T2–FLAIR mismatch sign in patients with LGG tumors, but also to evaluate the presence of this sign in high-grade tumors as well in a Greek cohort. Moreover, our main purpose was to evaluate if this sign could be a preoperative predictor of the patients’ IDH status.

## 2. Materials and Methods

### 2.1. Study Design

In our retrospective study, we evaluated 24 consecutive adult patients with gliomas comparing the presence of the T2–FLAIR mismatch sign on their MRIs in relation to the co-existence of an IDH mutation. The hospital’s Institutional Review Board approved our study. The handling of all personal data was according to the World Medical Association, the Declaration of Helsinki, and the current Health Insurance Portability and Accountability Act regulations. Due to the retrospective nature of our study and the use of anonymized hospital data, no written consent was obtained from the study participants. The enrollment period extended from August 2017 to August 2020.

### 2.2. Eligibility Criteria

Adult patients with a newly diagnosed supratentorial intra-axial brain tumor, free of calcifications and/or hemorrhagic products, who underwent resective surgery in our institution, and had a histopathological and genetic analysis of the resected tumor were included in our study. We focused on WHO Grade II, III, and IV gliomas with the following histologic diagnoses: astrocytoma, oligodendroglioma, oligoastrocytoma, diffuse glioma, and glioblastoma. Patients with infratentorial tumors were excluded from our current study, since there is no experience regarding the T2–FLAIR mismatch sign in such tumors. Likewise, patients with hemorrhagic, calcified, or recurrent gliomas were excluded due to the heterogeneity of the tumor texture. Finally, patients with incomplete medical records were excluded due to lack of critical data for our study.

### 2.3. Histological Diagnosis and Molecular Typing

A specialized neuropathologist performed the histologic assessment for all patients. Testing for IDH1/2 mutation by real-time PCR had been performed on samples received during the enrollment period [20]. 1p/19q co-deletions were detected using Multiplex Ligation-dependent Probe Amplification analysis on samples of paraffin-embedded tumor tissues [21].

### 2.4. Imaging Protocol

The MRI was performed at our institution on a 3T GE MRI scanner (GE HDx, Milwaukee, WI, USA) using an 8-channel neurovascular coil. All patients underwent 3D T1 weighted images (WI) pre- and post-contrast, axial T2 WI, axial T2 WI, FLAIR, diffusion WI, and T2* WI. Two neuroradiologists, with 15 and 2 years of experience, who were both blinded to the histological diagnosis, independently reviewed all images. The MRI images were evaluated for the presence (or absence) of the T2–FLAIR mismatch sign. The following parameters were recorded (i) location and laterality of the lesion, (ii) presence of midline shift, (iii) homogeneity/inhomogeneity of the lesion, (iv) peritumoral edema and mass effect, and (v) contrast enhancement. The following parameters were recorded: (i) location and laterality of the lesion, (ii) presence of midline shift, (iii) homogeneity/inhomogeneity of the lesion, (iv) peritumoral edema and mass effect, and (v) contrast enhancement. In the case of disagreement, the two raters reached a consensus after discussion.

### 2.5. Statistical Analysis

The basic characteristics of the study sample were summarized using the mean and standard deviation or counts and percentages for continuous and discrete parameters, respectively. The agreement between the two raters was estimated using Cohen’s kappa estimate. The effect of the T2–FLAIR mismatch sign on a number of parameters was estimated using univariate and multivariate logistic regression analysis. Of note, the multivariate analysis included parameters with a statistical significance of <0.05 in the univariate analysis. The results were summarized in odds ratios and marginal estimates of probabilities, along with their 95% confidence intervals (95% CI). Finally, we assessed the diagnostic accuracy of all models to predict the IDH gene status using a receiver operating characteristic (ROC) curve analysis. The diagnostic accuracy was described in terms of overall accuracy, specificity, sensitivity, and area under the curve (AUC).

## 3. Results

### 3.1. Study Sample Description

Our study sample included 24 patients with a mean age of 53.05 years (SD 12.84 years). Eleven (45.8%) were female (Table 1). Most lesions (*n* = 17, 70.8%) were located in the right hemisphere and on the frontal lobe (*n* = 16, 62.5%) in particular. The lesions were homogeneous in 14 cases (58.3%) and 11 cases (45.8%) were enhanced vividly after contrast administration. However, they were associated with minimal or no edema in more than half of the cases (*n* = 14, 58%) and midline shift on six occasions (25%). According to the 2016 WHO classification for CNS tumors [2], there were 15 (62.5%) low-grade and 9 (37.5) high-grade tumors in our study sample (Table 2). An IDH1/2 mutation was recognized in 15 instances (62.5%) within our cohort.

Kappa = 0.647 (95% CI: 0.337–0.957).
Kappa interpretation:
Kappa < 0: no agreement.
Kappa between 0.00 and 0.20: slight agreement.
Kappa between 0.21 and 0.40: fair agreement.
Kappa between 0.41 and 0.60: moderate agreement.
Kappa between 0.61 and 0.80: substantial agreement.
Kappa between 0.81 and 1.00: almost perfect agreement.

### 3.2. Agreement between the Two Reviewers

The first and the second raters recorded 16 (66.7%) and 14 (58.3%) occasions of theT2–FLAIR mismatch sign, respectively. The two raters agreed in 20 cases (Table 2). Cohen’s kappa coefficient was 0.647 (95% CI: 0.337–0.957). 

### 3.3. Logistic Regression

The univariate logistic regression identified that three parameters, including the homogeneity of the lesion (OR 14.0; 95% CI 1.86–106.27, *p* = 0.001), contrast enhancement (OR 0.0313; 95% CI 0.03–0.356, *p* = 0.002), and the T2–FLAIR mismatch sign (OR 52.0; 95% CI 4.03–670.6, *p* = 0.005) were independent predictors of the IDH status (Table 3). However, none of them retained its statistical significance in any multivariate model, except for the T2–FLAIR mismatch sign (OR 29.642; 95% CI 1.73–509.15, *p* = 0.019), when co-registered with homogeneity (Table 4).

### 3.4. Probabilities

**Figure 2:**
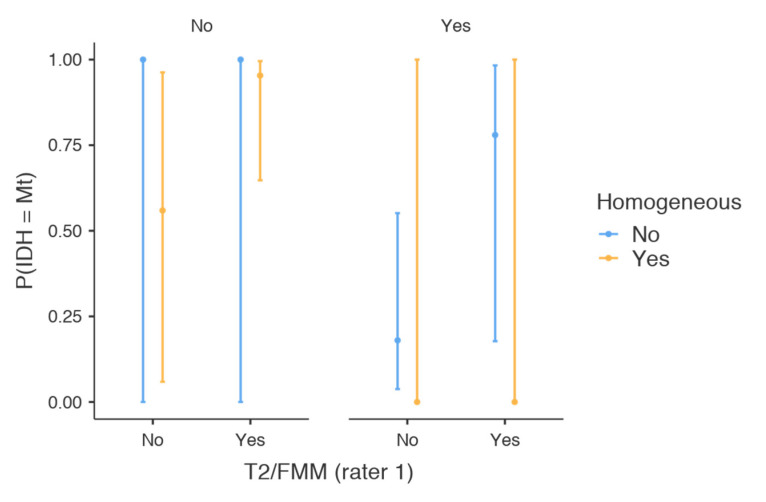
Marginal means plot of the full model. The probability of being IDH mutant in the presence of the T2–FLAIR mismatch sign and the absence of contrast enhancement was as high as 95.5% (95% CI 64.9–99.6%). Contrariwise, the probability of being IDH mutant in the absence of the T2–FLAIR mismatch sign with contrast enhancement was as low as 16.2% (95% CI 3.4–51.3%).

Accordingly, the highest probabilities of distinguishing between mutant and wild-type IDH variants with the use of MRI were achieved when considering the T2–FLAIR mismatch sign. The probability of being IDH mutant in the presence of the T2–FLAIR mismatch sign was as high as 92.9% (95% CI 63–99%). On the contrary, the probability of being Mt in the absence of the T2–FLAIR mismatch sign dropped to 20% (95% CI 5.04–54.1%). It was only the co-registration of the contrast enhancement properties of the lesion that improved our discrimination between the two alleles. More specifically, the probability of being Mt in the presence of the T2–FLAIR mismatch sign and the absence of contrast enhancement was as high as 95.5% (95% CI 64.9–99.6%). Meanwhile, the probability of being IDH mutant in the absence of the T2–FLAIR mismatch sign along with contrast enhancement was as low as 16.2% (95% CI 3.4–51.3%). The predictive characteristics of the full model are visualized in Figure 2, while the results from all potential combinations are depicted in Table 5. 

### 3.5. Diagnostic Accuracy

**Figure 3:**
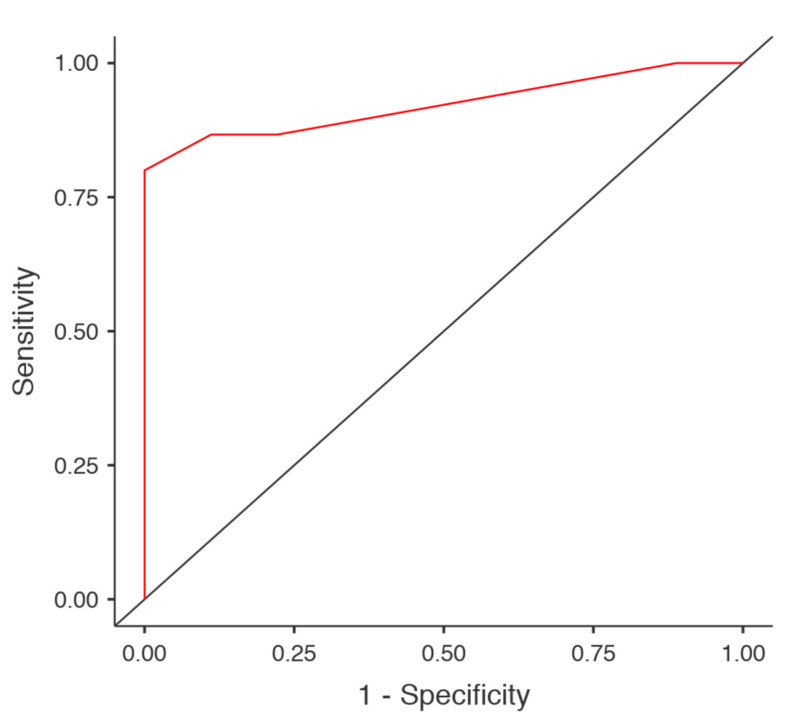
ROC curve of the full model. The full model was characterized by significant diagnostic accuracy (0.833) and very high AUC (0.922). ROC, receiver operating characteristic curve; AUC, area under the curve.

The sensitivity and specificity of the T2–FLAIR mismatch sign in the detection of the IDH mutation was 88.9% and 86.7%, respectively. The accuracy did not improve with the co-registration of any other parameter (Table 6). The highest AUC (0.922) was recorded in the full model (Figure 3).

## 4. Discussion

Our results showed that the T2–FLAIR mismatch sign represents a highly specific (86.7%) and sensitive (88.9%) imaging biomarker for the identification of IDH mutant gliomas. The recognition of this biomarker was helpful in distinguishing gliomas with a better prognostic profile, and significantly contributes in the therapeutic management of these patients. In addition, the T2–FLAIR mismatch sign constitutes an easily identifiable marker with substantial agreement between more and less experienced radiologists. Other important markers of the IDH status, but less accurate, were the extent of homogeneity within the lesion, as well as the degree of contrast enhancement after intravenous contrast administration.

Until recently, the T2–FLAIR mismatch sign has been used and validated for the prediction of 1p/19q status in IDH mutant LGGs [11,14,15,22]. In their pioneering study, Patel et al. described a positive correlation between the T2–FLAIR mismatch sign and the absence of 1p/19q codeletion in IDH mutant LGGs, with estimated positive (PPV) and negative predictive (NPV) values as high as 100% and 54%, respectively [11]. However, they mentioned that even though it is highly specific for LGGs, they have not evaluated the presence of this mismatch in other non-astrocytic low-grade tumors [11]. In another study, Lasocki et al. found that the presence of T2–FLAIR mismatch over 50% was highly predictive of a non-codeleted tumor. Similarly, Broen et al. validated the diagnostic accuracy of the T2–FLAIR mismatch sign in a selected population of adults with supratentorial molecularly defined LGG cases from three tumor registries [14]. The authors postulated that the sensitivity and specificity of the T2–FLAIR mismatch sign were as high as 51% and 100%, respectively [14]. Batchala et al. proposed a two-step classification algorithm based on neuroimaging metrics and the patient’s age, which demonstrated a moderate prediction accuracy of 1p/19q status in IDH mutant LGGs [22]. The first step of the algorithm was based on an assessment of the T2–FLAIR mismatch sign. In positive cases, the predictive accuracy for the existence of the IDH mutant 1p/19q non-codeleted subtype was high [22]. In the absence of the T2-FLAIR–mismatch sign, a model based on the tumor’s texture, the patient’s age, the presence of T2* blooming, the tumor’s location, and the presence of hydrocephalus was used instead [22]. The prediction accuracies of the algorithm were 81.1% and 79.2% in two independent readers [22].

Early last year, Juratli et al. studied the correlation of the T2–FLAIR mismatch sign and an array of molecular patterns in patients with WHO Grade II and III gliomas [23]. The authors classified 133 patients from two tumor databases in three groups, according to the molecular characteristics of the tumors: Group O (IDH mutant, 1p/19q codeleted oligodendrogliomas), Group A (IDH mutant, ATRX inactivated astrocytomas), and Group G (IDH wild-type, GBM-like) [23]. The prevalence of the T2–FLAIR mismatch sign was as high as 28.5%, 73%, and 0% in Groups O, A, and G, respectively [23]. Of note, patients in Groups A and O had a longer progression-free survival than those in Group G [23]. Subsequently, Jain et al. provided a number of key points for the proper use of the T2–FLAIR mismatch sign and suggested that the sign under study could be used for the exclusion of IDH wild-type gliomas [16]. Indeed, Foltyn et al. recognized the T2–FLAIR mismatch sign in 12 of 113 cases (10.6%) (Grade II and III gliomas) and in none of the 295 glioblastoma cases [19]. A recent meta-analysis by Goyal et al. estimated the pooled accuracy of the T2–FLAIR mismatch sign in predicting IDH mutation based on data extracted from three studies focusing on the prediction of 1p/19q status in patients with LGG [11,14,15,24,25]. The pooled sensitivity and specificity rates were estimated to be as high as 31% and 100%, respectively. Moreover, Deguici et al. reported that the T2–FLAIR mismatch sign was found in 45% of the 22 patients with IDH mutant astrocytoma and in only 5% of those with oligodendroglioma or IDH wild-type astrocytoma [18]. In the latter study, the positive predictive value of the T2–FLAIR mismatch sign was as high as 83% [18]. Throckmorton et al. claimed that broadening the criteria to include T2-heterogeneous LGG lesions increased the sensitivity of the T2–FLAIR mismatch sign by 30% [26]. According to a population-based study focusing on LGGs by Correl et al., the sensitivity and specificity of the mismatch sign for IDH mutation detection were 26.4% and 97.6%, respectively [27]. Our study extended the spectrum of glioma to include patients with high-grade glioma (HGG). Thus, we included a mixed population of patients with both LGG and HGG lesions in our study. We detected the T2–FLAIR mismatch sign in 13 of the 16 cases (81.3%) with LGG and in none of those with HGG. Thus, it seems that the T2–FLAIR mismatch sign is characterized by a high predictive value for the presence of an IDH1/2 mutation. In other words, it constitutes a robust tool to identify lesions with an improved prognostic profile among patients with supratentorial, diffuse, and infiltrating lesions.

The current study was characterized by some important limitations. Our results come from a small sample study. Further high-quality studies with a larger study sample are necessary for validating our current results. In addition, we did not include patients with IDH mutant anaplastic gliomas and IDH mutant glioblastomas (formerly known as secondary anaplastic gliomas and secondary glioblastoma) in our study. Thus, we could not control the performance of the T2–FLAIR mismatch sign for these particular patient subgroups. Equally important, our study excluded pediatric patients and thus we could not study the role of the T2–FLAIR mismatch sign in pediatric gliomas. However, Johnson et al. reported five cases with false positive T2–FLAIR mismatch signs occurring outside the context of IDH mutant astrocytomas, predominantly in children or young adults with pediatric-type gliomas [28]. Among them, there was a case of a 44-year-old adult with an IDH mutant and 1p/19q codeleted oligodendroglioma [28]. Furthermore, it has to be pointed out that the interobserver agreement was low in our study. The T2–FLAIR mismatch sign is based on qualitative imaging MRI analysis. Therefore, there is an existing risk for misclassification bias, particularly by less experienced observers. That is exactly the reason why advanced MRI, based on robust quantitative data, is required.

## 5. Conclusions

The MRI constitutes a useful tool in glioma subtyping. The T2–FLAIR mismatch sign seems to be an easy to use and helpful tool in recognizing IDH mutant gliomas preoperatively and also in cases where molecular testing is unavailable. This is particularly important, as suggested by the newly proposed WHO 2020 classification. We suggest the adoption of a protocol based on imaging and histological data for optimal glioma characterization.

## Figures and Tables

**Table 1:** Basic characteristics of our patient sample (*n* = 24).

		Mean	SD
Age (years)		53	12.8
	Subgroup	Counts	%
Gender	Female	11	45.8
	Male	13	54.2
Laterality	Left	7	29.2
	Right	17	70.8
Location	Frontal	15	62.5
	Temporal	5	20.8
	Insular	2	8.4
	Paracentral	1	4.2
	Parietal	1	4.2
Midline shift	No	18	75
	Yes	6	25
Homogeneous	No	10	41.7
	Yes	14	58.3
Edema	No	10	41.7
	Minimal	4	16.7
	Yes	8	33.3
	Significant	2	8.3
Contrast enhancement	No	13	54.2
	Yes	11	45.8
IDH status	Mutant type	15	62.5
	Wild-type	9	37.5
WHO classification of the CNS tumors	Diffuse astrocytoma, IDH mutant	12	50
	Anaplastic astrocytoma, IDH wild-type	1	4.2
	Glioblastoma, IDH wild-type	8	33.3
	Oligodendroglioma, IDH mutant and 1p/19q codeleted	2	8.4
	Oligodendroglioma, NOS	1	4.2

**Table 2:** Contingency table of the two rates regarding the T2–FLAIR mismatch sign.

		Observer 2		
		Yes	No	Total
Observer 1	Yes	13	1	14
	No	3	7	10
	Total	16	8	24

Number of observed agreements: 20 (83.33% of the observations).

**Table 3:** Univariate logistic regression. The isocitrate dehydrogenase (IDH) status could be predicted based on the homogeneity, contrast enhancement, and the T2–FLAIR mismatch sign.

			Univariate Logistic Regression	
		Reference	OR (95% CI)	*p*
Laterality	Right	Left	1.37 (0.228–8.30)	0.728
Location	Insular	Frontal	1.14 × 10^8^ (0–Inf)	0.998
	Paracentral	Frontal	1.14 × 10^8^ (0–Inf)	0.999
	Parietal	Frontal	1.14 × 10^8^ (0–Inf)	0.999
	Temporal	Frontal	1.16 × 10^−9^ (0–Inf)	0.997
Midline shift	Yes	No	6.36 × 10^−10^ (0–Inf)	0.996
Homogeneous	Yes	No	**14.0 (1.86–106.27)**	**0.01**
Edema	Minimal	No	9.54 × 10^−9^ (0–Inf)	0.996
	Significant	No	1.01 × 10^−17^ (0–Inf)	0.996
	Yes	No	1.06 × 10^−9^ (0–Inf)	0.995
CE	Yes	No	**0.0313 (0.03–0.356)**	**0.005**
T2–FLAIR mismatch sign	Yes	No	**52.0 (4.03–670.6)**	**0.002**

OR, odds ratio; CI, confidence interval; CE, contrast enhancement, Inf, infinity. Note. The OR represents the odds ratio of “IDH = mutant” vs. “IDH = wild-type”. Bold highlight statistical significance.

**Table 4:** Multivariate logistic regression analysis of four models. No parameter retained its statistical significance in any multivariate model, except for the T2–FLAIR mismatch sign when co-registered with tumor homogeneity.

			Multivariate Model 1		Multivariate Model 2		Multivariate Model 3		Multivariate Model 4	
		Reference	OR (95% CI)	*p*	OR (95% CI)	*p*	OR (95% CI)	*p*	OR (95% CI)	*p*
Homogeneous	Yes	No	5.48 × 10^−8^ (0–Inf)	0.997	2.802 (0.177–44.29)	0.464	-	-	0.07 × 10^−7^ (0–Inf)	0.997
CE	Yes	No	1.96 × 10^−9^ (0–Inf)	0.996	-	-	0.161 (0.007–3.37)	0.239	1.85 × 10^−8^ (0–Inf)	0.996
T2–FLAIR mismatch sign	Yes	No	-	-	**29.642 (1.73–509.15)**	**0.019**	17.585 (0.919–336.55)	0.057	16.1 (0.824–315)	0.067

Note. The OR represents the odds ratio of “IDH = mutant” vs. “IDH = wild-type”. Multivariate Model 1: IDH ~ CE + T2–FLAIR mismatch sign; Multivariate Model 2: IDH ~ homogeneous + T2–FLAIR mismatch sign; Multivariate Model 3: IDH ~ homogeneous + CE; Multivariate Model 4: IDH ~ Homogeneous + CE + T2–FLAIR mismatch sign. Bold highlight statistical significance.

**Table 5:** Estimated marginal means table. Probability of IDH mutation in association with various radiological parameters.

					95% Confidence Interval	
Contrast Enhancement	Homogeneous	T2–FLAIR Mismatch Sign	Probability	SE	Lower	Upper
Univariate Model 1						
	No		0.3	0.1449	0.0998	0.624
	Yes		0.857	0.0935	0.5732	0.964
Univariate Model 2						
		No	0.2	0.1265	0.0504	0.541
		Yes	0.929	0.0688	0.6297	0.99
Univariate Model 3						
No			0.923	0.0739	0.6094	0.989
Yes			0.273	0.1343	0.0905	0.586
Multivariate Model 1						
No		No	0.545	0.3795	0.0562	0.96
		Yes	0.955	0.0537	0.649	0.996
Yes		No	0.162	0.1174	0.0341	0.513
		Yes	0.772	0.2489	0.1748	0.982
Multivariate Model 2						
	No	No	0.162	0.1231	0.0317	0.533
		Yes	0.852	0.1846	0.2469	0.99
	Yes	No	0.352	0.2913	0.0424	0.869
		Yes	0.941	0.0615	0.6434	0.993
Multivariate Model 3						
No	No		1	1.81 × 10^−5^	2.22 × 10^−16^	1
Yes	No		0.3	0.1449	0.0998	0.624
No	Yes		0.923	0.0739	0.6094	0.989
Yes	Yes		2.35 × 10^−8^	9.30 × 10^−5^	2.22 × 10^−16^	1
Multivariate Model 4						
No	No	No	1	3.34 × 10^−4^	2.22 × 10^−16^	1
		Yes	1	2.07 × 10^−5^	2.22 × 10^−16^	1
	Yes	No	0.559	0.37852	0.0589	0.963
		Yes	0.953	0.05465	0.6475	0.996
Yes	No	No	0.18	0.12978	0.0377	0.552
		Yes	0.78	0.24513	0.1775	0.983
	Yes	No	2.35 × 10^−8^	9.30 × 10^−5^	2.22 × 10^−16^	1
		Yes	3.79 × 10^−7^	0.0015	2.22 × 10^−16^	1

Univariate Model 1: IDH ~ homogeneity; Univariate Model 2: IDH ~ T2–FLAIR mismatch sign; Univariate Model 3: IDH ~ contrast enhancement; Multivariate Model 1: IDH ~ CE+ T2–FLAIR mismatch sign; Multivariate Model 2: IDH ~ homogeneous + T2–FLAIR mismatch sign; Multivariate Model 3: IDH ~ homogeneous + CE; Multivariate Model 4 ~ IDH Homogeneous + CE + T2–FLAIR mismatch sign.

**Table 6:** **Diagnostic accuracy table.** Sensitivity and specificity in predicting the IDH status were the highest when the T2–FLAIR mismatch sign and homogeneity were co-registered.

	Parameters	Accuracy	Specificity (95% CI)	Sensitivity (95% CI)	AUC
Univariate	T2–FLAIR mismatch sign	0.875	0.867 (0.584–0.976)	0.889 (0.507–0.994)	0.878
	CE	0.833	0.800 (0.513–0.947)	0.889 (0.507–0.994)	0.844
	Homogeneity	0.792	0.800 (0.513–0.947)	0.778 (0.402–0.960)	0.789
Multivariate	CE, Homogeneity	0.833	0.800 (0.513–0.947)	0.889 (0.507–0.994)	0.856
	T2–FLAIR mismatch sign, CE	0.833	0.867 (0.584–0.976)	0.778 (0.402–0.960)	0.915
	T2–FLAIR mismatch sign, Homogeneity	0.875	0.867 (0.584–0.976)	0.889 (0.507–0.994)	0.907
	T2–FLAIR mismatch sign, CE, Homogeneity	0.833	0.867 (0.584–0.976)	0.778 (0.402–0.960)	0.922

CE, contrast enhancement; AUC, area under the curve.
